# The crosstalk of CD8+ T cells and ferroptosis in cancer

**DOI:** 10.3389/fimmu.2023.1255443

**Published:** 2024-01-15

**Authors:** Zhengjun Lin, Songzhu Zou, Kunming Wen

**Affiliations:** ^1^ Department of General Surgery, Affiliated Hospital of Zunyi Medical University, Zunyi, Guizhou, China; ^2^ The First People's Hospital of Zunyi, The Third Affiliated Hospital of Zunyi Medical University, Zunyi, Guizhou, China

**Keywords:** CD8+ T cells, IFN-γ, ferroptosis, DAMPs, immunotherapy

## Abstract

Ferroptosis is an iron-dependent, novel form of programmed cell death characterized by lipid peroxidation and glutathione depletion and is widespread in a variety of diseases. CD8+ T cells are the most important effector cells of cytotoxic T cells, capable of specifically recognizing and killing cancer cells. Traditionally, CD8+ T cells are thought to induce cancer cell death mainly through perforin and granzyme, and Fas-L/Fas binding. In recent years, CD8+ T cell-derived IFN-γ was found to promote cancer cell ferroptosis by multiple mechanisms, including upregulation of IRF1 and IRF8, and downregulation of the system XC-, while cancer cells ferroptosis was shown to enhance the anti-tumor effects of CD8+ T cell by heating the tumor immune microenvironment through the exposure and release of tumor-associated specific antigens, which results in a positive feedback pathway. Unfortunately, the intra-tumoral CD8+ T cells are more sensitive to ferroptosis than cancer cells, which limits the application of ferroptosis inducers in cancer. In addition, CD8+ T cells are susceptible to being regulated by other immune cell ferroptosis in the TME, such as tumor-associated macrophages, dendritic cells, Treg, and bone marrow-derived immunosuppressive cells. Together, these factors build a complex network of CD8+ T cells and ferroptosis in cancer. Therefore, we aim to integrate relevant studies to reveal the potential mechanisms of crosstalk between CD8+ T cells and ferroptosis, and to summarize preclinical models in cancer therapy to find new therapeutic strategies in this review.

## 1 Introduction

CD8+ T cells can resist tumor growth and metastasis through various mechanisms (1–3). Firstly, when normal cells transform into cancer cells, they express specific antigens. CD8+ T cells directly attack cancer cells by recognizing these specific antigens and inhibit or slow down tumor growth (4, 5). Secondly, the exposure and release of tumor antigens can activate other immune cells to directly or indirectly promote the anti-tumor effects of CD8+ T cells (6, 7). However, cancer cells can also evade the attack of CD8+ T cells through various strategies, such as reducing the expression of tumor-specific antigens, increasing infiltration of immune suppressor cells, blocking the activation of CD8+ T cells, inducing the exhaustion and death of CD8+ T cells (8–13). Therefore, the study is a hot topic about how to enhance the immune response of CD8+ T cells against tumors and overcome the escape mechanisms of cancer cells in the field of cancer immunotherapy.

Ferroptosis was proposed in 2012, referring to a distinct form of programmed cell death triggered by iron-dependent and lipid peroxidation pathways, which is widely present in various diseases, especially cancer (14–18). In recent years, the mechanisms of ferroptosis have been rapidly elucidated, including system XC- inhibition (SLC7A11/SLC3A2), upregulation of glutathione peroxidase 4 (GPX4), iron homeostasis imbalance, and phospholipid peroxidation (14, 19–22). Multiple factors can also regulate the sensitivity of cell ferroptosis in pathological conditions to mediate disease progression (23). The system XC- is a reverse transporter of cystine and glutamate, which can increase cellular uptake of cystine and convert it to glutathione (GSH) under the action of thioredoxin reductase 1 (TXNRD1), and GPX4 relies on GSH as a substrate to increase its activity and promote the conversion of lipid peroxide (PL-OOH) to lipid alcohol (PL-OH). Thus, It prevents cells ferroptosis by decreasing the accumulation of PL-OOH. Erastin and RSL3 act as inhibitors of the system XC- and GPX4, respectively, to promote cell ferroptosis (14, 19, 23). Iron homeostasis imbalance is another classical pathway in ferroptosis, regulated mainly by the network of transferrin receptor 1 (TfR1), iron regulatory protein 1 (IRP1), and iron regulatory protein 2 (IRP2) to influence cellular iron uptake, storage, and release. The production of reactive oxygen species (ROS) and phospholipid peroxidation require a large amount of metabolic enzyme involvement and iron acts as a catalyst and essential element for these enzymes. Iron-dependent Fenton reaction rapidly amplifies PL-OOHs and produces various reactive free radicals to induce cancer cell ferroptosis (24–26). Infinite lipid peroxidation is a hallmark of ferroptosis, and acyl-CoA synthetase long-chain family member 4 (ACSL4) is a key enzyme in the conversion of polyunsaturated fatty acids (PUFAs) to PUFA-PE. PUFA-PE promotes the intracellular accumulation of lipid peroxides under the action of various enzymes. The cell membrane contains abundant PUFA-PL, and phospholipid peroxidation is considered the direct executor of cell ferroptosis (27–29).

In recent years, it has been found that CD8+ T cells- derived IFN-γ promotes cancer cells ferroptosis, which can release multiple tumor antigens and further activate CD8+ T cells through the role of antigen-presenting cells (APCs) to enhance anti-cancer immunity. In addition, CD8+ T cells and other immune cells can undergo ferroptosis in the tumor microenvironment (TME), thereby altering the immune function of CD8+ T cells in tumors. Therefore, this review introduces mainly the network of CD8+ T cells and ferroptosis in cancer and reveals the underlying mechanisms.

## 2 CD8+ T cells ferroptosis causes immune escape of cancer cells

CD8+ T cells are the most effective immune cells in anti-cancer immunity and can directly kill cancer cells in multiple ways, earning them the nickname “executioner” of the tumor immune system (30). Perforin and granzyme released by CD8+ T cells are effector factors of cancer cell apoptosis, which can lead to cancer cell protein degradation and destruction, causing cell apoptosis (31). Another way is through CD8+T cells Fas-L binding to target cell Fas, sequentially activating caspase 8 and caspase 3 proteases to promote protein degradation, causing lethal damage to cancer cells (32–34). CD8+ T cells can also indirectly kill cancer cells by releasing cytokines such as tumor necrosis factor (TNF) (35). In recent years, it has been found that CD8+ T cells-derived IFN-γ can induce cancer cells ferroptosis by binding to the surface IFN- γ receptor (IFNγR) on cancer cells, which enriches the mechanisms of CD8+ T cell killing cancer cells (36–39). Ferroptosis plays a crucial role in CD8+ T cell-mediated anti-tumor immunity. However, CD8+ T cells are also vulnerable in the TME. Cancer cells upregulate molecules such as PD-L1 and Fas-L to cause the dysfunction and exhaustion of CD8+ T cells and promote immune evasion of cancer cells (9, 40–43). New evidence also suggests that cancer cells induce CD8+ T cell ferroptosis by interfering with the TME, weakening their anti-cancer immune function (44).

Recent studies have shown that CD8+ T cells are more sensitive to ferroptosis than cancer cells and are susceptible to spontaneous ferroptosis influenced by the TME, depriving survival opportunities of CD8+ T cells. This leads to a decrease in the abundance of intra-tumoral CD8+ T cells and induces functional impairment, promoting immune evasion of cancer cells. Drijvers et al. demonstrated that activated CD8+ T cells were significantly more sensitive to RSL3-induced ferroptosis than cancer cells when CD8+ T cells were co-cultured with cancer cells, resulting in ferroptosis of CD8+ T cells and immune regression against cancer. Lack of ACSL4 protected CD8+ T cells from the threat of high-dose RSL3-induced ferroptosis but also resulted in functional defects of CD8+ T cells, enabling cancer cells to evade the specific killing of CD8+ T cells. Therefore, the key enzyme ACSL4, which regulates lipid peroxidation during cell ferroptosis, is necessary to maintain the function of CD8+ T cells (44). In addition, the sensitivity of CD8+ T cell ferroptosis is also influenced by the metabolic state of the TME. Ma et al. found that cholesterol is rich in the TME and gradually increases with tumor progression. Cholesterol induces the expression of CD36 on the intra-tumoral CD8+ T cells. CD36 can increase intracellular fatty acid uptake and lipid accumulation to trigger lipid peroxidation-induced ferroptosis and weaken the anti-cancer ability of CD8+ T cells. This explains why the gradual upregulation of CD36 expression reduces the infiltration of intra-tumoral CD8+ T cells through ferroptosis during tumor progression, leading to a gradual decline in anti-tumor immune function (45, 46). Conversely, CD8+ T cells can also evade ferroptosis by adjusting the expression of ferroptosis-related genes. Tc1 is a typical subset of CD8+T cells, and Tc9 is a subset of CD8+T cells that secrete IL-9 (47). Xiao et al. found Tc9 cells transferred into tumor-bearing mice exhibit longer lifespan and anti-tumor activity than Tc1 cells, thanks to the high expression of IL-9 in Tc9 cells. STAT3 is a downstream target of IL-9 and can bind to the CPT1A promoter to induce transcription. CPT1A, as a key enzyme in fatty acid oxidation, can increase mitochondrial activity and reduce lipid peroxidation, thereby protecting Tc9 cells from tumor-induced ferroptosis. In melanoma, the IL-9 expression is lower and the expression of genes related to lipid peroxidation and ferroptosis is higher on the tumor Infiltrating CD8+ T cells, compared with circulating CD8+ T cells, which may be related to increased sensitivity of the intra-tumoral CD8+ T cells to ferroptosis (48). It is worth noting that IL-9 has been shown to activate adaptive immune to suppressive tumor growth in various tumors (49), but it has a tumor-promoting effect in T cell-derived hematological cancers (50, 51). It needs further investigation whether high expression of IL-9 is involved in protecting T cell-derived hematological cancer cells from ferroptosis.

The high sensitivity of CD8+ T cells to ferroptosis in the TME limits the application of ferroptosis inducers in cancer and increases the immune evasion of cancer cells. By further studying CD8+ T cells and identifying protective factors that prevent them from undergoing ferroptosis, it may be possible to enhance the effectiveness of anti-tumor immune therapy.

## 3 The interaction of CD8+ T cells and cancer cells ferroptosis enhances tumor suppression

**Figure 1:**
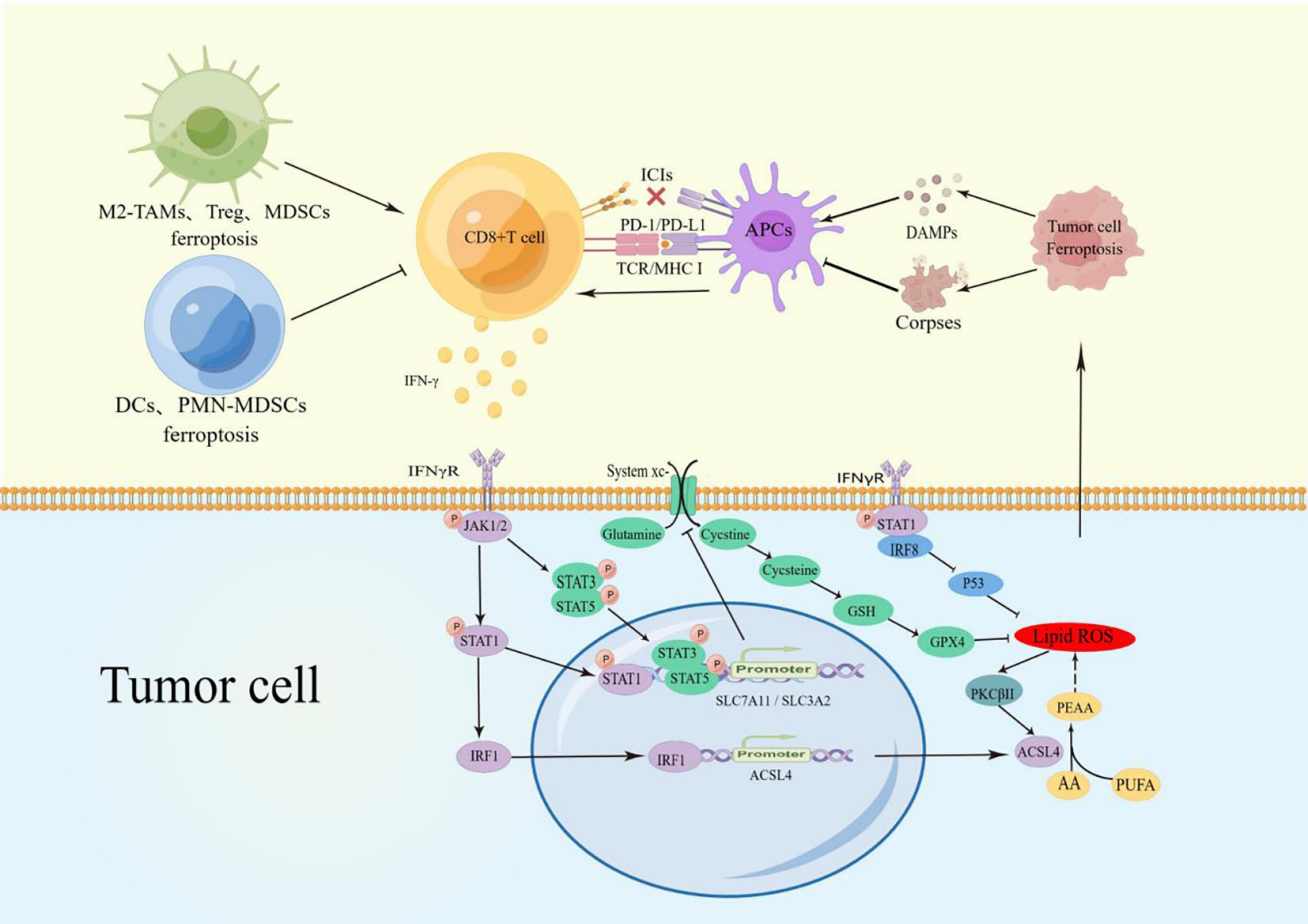
TAMs Ferroptosis reduced M2-macrophage infiltration and increased polarization to M1 macrophages to promote CD8+ T cell activity. Treg, MDSCs, and TINs ferroptosis reduced the inhibition of CD8+ T cells. PMN-MDSCs and DCs blocked the CD8+ T-cell activation pathway.CD8+ T cell-derived IFN-γ binds to IFNγR on the surface of cancer cells and promotes phosphorylation of STATs via JAK1/2. P-STAT1 promotes ACSL4 expression through upregulation of IRF1, and the combination of ACSL4 and AA can induce cell ferroptosis. During ferroptosis, the increased lipid ROS can activate PKCβII and ACSL4 to Significantly magnify this effect. P-STAT1, p-STAT3, and p-STAT5 inhibits SLC7A11/SLCA2 expression to block the uptake of cystine and reduce the activity of GPX4, which also promotes cells ferroptosis. In addition, IFNγR can up-regulate the expression of IRF8. These factors together amply tumor cell ferroptosis. The release of DAMPs during cancer cell ferroptosis induces the maturation of APCs and activation of CD8+ T cells, establishing a positive recycling pathway. Due to engulfing ferroptosis cell corpses, APCs lose their antigen-presenting ability and have difficulty activating CD8+T cells.

The interaction between CD8+ T cells and cancer cell ferroptosis is mutually promoting. The CD8+ T cells-derived IFN-γ induces cancer cell ferroptosis by binding to IFNγR and activating multiple pathways. Cancer cell ferroptosis releases various tumor antigens that activate CD8+ T cells through APCs, forming a “positive closed-loop pathway” that significantly enhances tumor suppression (**Figure 1**).

### 3.1 CD8+ T cells promote cancer cell ferroptosis

IFN-γ is one of the effector factors secreted by activated CD8+ T cells and exerts its function by binding to IFNγR on cancer cells. STATs and interferon regulatory factors (IRF) family are mainly downstream genes of IFN-γ (52). In CD8+ T cell-induced cancer cell ferroptosis, IFN-γ regulates the phosphorylation of STATs (P- STATs) and changes the transcriptional activation of target genes. P-STATs mediate the expression of IRF1 and IRF8 in cancer cells (53, 54), which act as the transcriptional activation factors of ACSL4 and the transcriptional inhibitory factors of P53, respectively, to participate in the induction of cancer cell ferroptosis (36, 55). The low expression of IRF8 in hepatocellular carcinoma and breast cancer has been associated with a low response to immune therapy (IFN-γ and ICIs). In addition, IFN-γ is involved in mitochondrial damage and cell cycle arrest, further promoting cancer cell ferroptosis.

With the action of IFN-γ, the activation of JAK1/2 and phosphorylation of STAT1 increase the expression of IRF1 on cancer cells. IRF1 acts as a transcriptional activation factor on the ACSL4 promoter. ACSL4 is a key enzyme for fatty acid oxidation and reshapes the lipid spectrum in cancer cells under the action of arachidonic acid (AA), increasing lipid peroxidation levels and ferroptosis. Therefore, IFN-γ combined with AA is thought as the endogenous triggering factor for ACSL4-mediated cancer cell ferroptosis, and immune checkpoint inhibitors (ICIs) can significantly enhance the anti-tumor effect (36). Interestingly, recent studies have shown that the accumulation of lipid peroxides induces the activation of PKCβII in cancer cells, and PKCβII can also interact with the Thr328 site of ACSL4 to activate ACSL4. Therefore, the combined use of IFN-γ and fatty acids may promote the rapid amplification of lipid peroxides through positive feedback between PKCβII and ACSL4, reaching a lethal level of cancer cell ferroptosis and enhancing the ability of IFN-γ to induce ferroptosis (56). In another study, Wang et al. found that IFN-γ-mediated P-STAT1 can directly bind to the promoter region to inhibit the expression of SLC7A11 and increase significantly intracellular GSH depletion, lipid peroxidation, and ferroptosis in cancer (37). Consistent with the above studies, Kong et al. verified that IFN-γ treatment induces cancer cell ferroptosis through the STAT1/IRF1/ACSL4 axis in hepatocellular carcinoma. In addition, they found that IFN-γ increases the phosphorylation of STAT3 and inhibits the transcription of SLC7A11, further reducing cystine uptake, and disrupting intracellular redox balance. They also found that the combination of IFN-γ and Erastin enhances mitochondrial oxidation and the loss of mitochondrial membrane potential (MMP), further triggering ferroptosis. Moreover, the combination of IFN-γ and ferroptosis inducers can inhibit the expression of CyclinD1, CDK4, and CDK6 to mediate more cell cycle arrest (39). It has been reported that phosphorylated STAT5 can also bind to the promoter region of SLC7A11 to inhibit its expression, participating in cystine deprivation and disrupting intracellular redox balance, which may trigger ferroptosis (57). IRF8 is another target gene of IFN-γ. Poschel et al. found that the expression level of IRF8 is significantly higher in responders to nivolumab (PD-1 inhibitor) treatment in melanoma patients compared to non-responders. IRF8 can inhibit the expression of P53 protein, increase lipid peroxidation, and promote ferroptosis in melanoma cells (55). Similarly, the IFN-γ and ICIs immune therapies signaling pathway is significantly inhibited with low levels of IRF8 in hepatocellular carcinoma and is associated with poor prognosis (58), while high expression of IRF8 in human breast cancer is associated with better response to immune therapy and chemotherapy (59). Whether IRF8 regulated by immune therapy interferes with ferroptosis is worth further investigation.

In summary, activated CD8+ T cells secrete IFN-γ and act on the key targets of cancer cell ferroptosis. This leads to a decrease in the oxidative buffering capacity of GPX4 and other GSH-dependent enzymes. IFN-γ also promotes lipid peroxidation through ACSL4 using fatty acids and results in selective enrichment of PUFAs to induce cells ferroptosis (38). Ferroptosis is usually induced by exogenous chemical molecules, including erastin, RSL3, and Sorafenib. CD8+ T cell-derived IFN-γ is an important component of anti-cancer immunity, and AA exists in plasma and cells, jointly opening the door to endogenous ferroptosis (36).

### 3.2 Cancer cell ferroptosis influences the antitumor ability of CD8+ T cell

The Cell Death Naming Committee has defined immunogenic cell death (ICD) as “a regulated form of cell death that is capable of activating adaptive immune responses in immunocompetent hosts of the same genotype” (60). Many studies have shown that various factors are exposed or released during cell death processes such as necrosis, pyroptosis, autophagy, and apoptosis, which present “find me” and “eat me” signals to mediate immune responses. These factors are collectively referred to as damage-associated molecular patterns (DAMPs) (61). Consistent with other death-related ICDs, Ferroptosis, as a novel form of programmed cell death, can also release DAMPs such as HMGB1, calreticulin, ATP, HSP70, and HSP90 during the death process. These factors are often simultaneously released by cancer cells and act as immune adjuvants to attract and stimulate antigen-presenting cells (APCs). The activated APCs can engulf and process tumor-associated antigens. This ultimately leads to the clonal expansion of tumor-specific CD8+ T cells and elicit an immune response. However, inconsistent with other ICDs, ferroptosis can also inhibit the activation of CD8 T cells due to lipids peroxidation, which causes lipid droplet accumulation and loss of antigen function after APCs engulf ferroptosis corpses (**Table 1**).

**Table 1:** Cancer cells ferroptosis influences CD8+ T cell immunity.

DAMPS	Ferroptosis induction	Effect	Mechanism	Refs
HMGB1	OTUD1	Promotion	Increase in intra-tumor CD8+ T cells	(62)
	ML266	Promotion	induce BMDCs maturation and activation of CD8+ T cells	(63)
	GPX4 deficiency	Inhibition	Promote infiltration of MDSCs	(64)
ATP	ML266	Promotion	induce BMDCs maturation and initiate CD8+ T cells	(63)
	SCD1 inhibition	Promotion	Induce ferroptosis immunogenicity, promote activation of DCs, and activate immunity.	(65)
	Shikonin	Promotion	release ATP and HMGB1, promote ICD	(66)
CRT	RSL3	Promotion	Promote the transfer of CRT to the cell surface and increase the number of CD8+ T cells	(67)
	HCSVs+MF	Promotion	Induce DCs maturation and cytotoxic T lymphocyte infiltration	(68)
Corpses	GPX4 inhibition	Inhibition	Impair antigen presentation function of DCs and inhibit the activation of CD8+ T cells	(69)

High mobility group box 1 (HMGB1) is a non-histone nuclear protein that has different functions depending on its subcellular localization. Extracellular HMGB1 regulates immune responses by binding to immune cell receptors (70, 71). In various diseases, HMGB1 acts as a redox protein that increases the accumulation of reactive oxygen species (ROS). ROS is considered a stress factor for ferroptosis, and the migration and release of HMGB1 are associated with ROS. Therefore, HMGB1 is often released during ferroptosis and participates in the regulation of CD8+ T cells as tumor antigens (72–77). Song et al. found that overexpression of OTUD1 inhibits the ubiquitination and degradation of iron-responsive element-binding protein 2 (IREB2) in colorectal cancer cells. IREB2, as an iron sensor, participates in the regulation of iron transport proteins and promotes iron uptake and ferroptosis in cancer cells, which significantly increases the release of HMGB1 in the tumor stroma and promotes the CD8+ T cells infiltration in cancer (62). In addition, Efimova et al. treated fibrosarcoma cells and glioblastoma cells with RSL3 and detected the immunogenic characteristics of ferroptosis cells at different time points. The release of HMGB1 reached its maximum level only in the late stage of ferroptosis (24h). The level of HMGB1 in the supernatant of early-stage ferroptotic cells (3h) showed no statistically significant difference compared to that in surviving cancer cells. However, when fibrosarcoma cells and glioblastoma cells were co-cultured with bone marrow-derived dendritic cells (BMDCs), the level of HMGB1 reached its maximum value in the early stage (3h). Whether BMDCs alter the spatiotemporal release of HMGB1 from ferroptotic cells is still unclear. However, early-stage ferroptotic cells can induce BMDCs maturation and active CD8+ T cells to suppress tumor growth by involving. Late-stage ferroptosis cells, on the other hand, can be cleared, but lack immunogenic characteristics (63).

It is worth noting that the role of HMGB1 in immunity is not solely to promote antigen presentation and activate CD8+ T cells. Some studies have shown that HMGB1 can increase the infiltration of immune suppressive cells to weaken the anti-cancer effects of CD8+ T cells (78). Conche et al. found that GPX4 deficiency induces lipid peroxidation and ferroptosis in hepatocellular carcinoma. During this process, it can recruit CD8+ T cells by increasing the expression of CXCL10 and PD-L1 in cancer cells. However, the increase of HMGB1 during ferroptosis leads to the infiltration of myeloid-derived suppressor cells (MDSCs), which are immune suppressive cells in the TME. Blocking the inhibitory effect of MDSCs on CD8+ T cells through ICIs can enhance the anti-cancer effects. The same treatment did not inhibit tumor growth in mice with colon cancer but reduced liver metastasis, indicating the complexity of ferroptosis-induced immunity in the different TMEs (64).

Adenosine triphosphate (ATP) is an essential nucleotide for metabolism and is released into the TME during cell death through lysosomal secretion and vesicle formation. It binds to immune cell receptors, such as DCs’ P2X7R receptor, to initiate tumor immune responses (79). In contrast to HMGB1, ATP is released by fibrosarcoma and glioma cells after 3 hours of RSL3 treatment, reaching a peak at 6 hours. However, the released ATP is depleted by 24 hours. This suggests that ATP induces DCs maturation and initiates CD8+ T cell immunity in the early stages of ferroptosis, but late-stage ferroptosis cells cannot acquire adaptive immunity. In esophageal cancer, inhibiting SCD1 under radiotherapy prevents the conversion of saturated fatty acids to unsaturated fatty acids, increases intracellular lipid peroxidation and ferroptosis, and activates DCs through recognition and activation of ATP released by cancer cells (65). Shikonin enhances ferroptosis and ATP release in multiple myeloma by inhibiting GOTI and promoting iron phagocytosis, thereby activating adaptive immunity (66).

Calreticulin (CRT) is an endoplasmic reticulum luminal Ca2+ buffering protein that is involved in regulating Ca2+ homeostasis and endoplasmic reticulum Ca2+ capacity (80). The damage of cancer cells triggers the gradual translocation and exposure of CRT as a “find me” signal. APCs, especially immature dendritic cells (DCs), can bind to CRT through the CD91 receptor to increase the activation of tumor-specific naive CD8+ T cells (81–84). Therefore, CRT is an important DAMP in the process of cell death. Cancer cell ferroptosis has also been shown to induce tumor immune responses through the exposure of CRT. Zhao et al. found a significant negative correlation between the expression of GPX4 and calreticulin in HNSCC. Inhibition of GPX4 by RSL3 significantly increased cancer cell ferroptosis, promoted the translocation of CRT to the cell surface, downregulated myeloid-derived suppressor cells and M2-like macrophages, increased the number of CD4+ T cells and CD8+ T cells, improved the immunosuppressive microenvironment of head and neck squamous cell carcinoma (HNSCC), and inhibited tumor progression (67). Similarly, Yu et al. used nanopolymer-mediated intracellular Fenton reaction and oxidative stress in cancer cells, which resulted in the exposure of CRT on ferroptotic cancer cells, leading to DCs maturation and infiltration of cytotoxic T lymphocytes (68).

Other DAMPs Cancer cell death is accompanied by the release of a large number of tumor antigenic molecules. In addition to HMGB1, CRT, and ATP mentioned above, there are many other antigenic factors involved in the process of ferroptosis, such as HSP70 and HSP90 (77). These factors usually accumulate in the TME, including but not limited to inducing the maturation and activation of M1-like macrophages or DCs, to amplify the immune response and enhance the anti-tumor ability of CD8+ T cells.

Contradictorily, recent reports have shown that cancer cell ferroptosis impedes DCs-mediated anti-tumor immunity, which challenges previous studies. ML266-induced cancer cell ferroptosis can release ATP and HMGB1, as well as expose CRT. However, when bone marrow-derived dendritic cells (BMDCs) were co-cultured with ferroptotic cancer cells, it was found that the maturation of BMDCs was negatively correlated with early-stage (1-2h) ferroptotic cancer cells. Although the maturation marker molecules of BMDCs increased in the mid-stage (3-4h) and late-stage (5-8h), the maturation of BMDCs was not sufficient to induce an immune response due to the loss of antigen presentation ability after BMDCs engulf ferroptosis cell corpse. this is manifested by the decrease of antigen presentation-related gene expression and the accumulation of lipid droplets. Thus, ferroptotic cancer cells weaken the ability to activate CD8+ T cells. The inoculation of ferroptotic cancer cells cannot induce tumor immunogenic protection against newly formed tumors *in vivo*, regardless of the stage of cell death (early, mid, or late stage) (69). This may explain the concept that ferroptotic cancer cells are associated with poor prognosis in various cancer patients (85–87).

In a word, CD8+T cells have a higher sensitivity to ferroptosis than cancer cells, and ferroptosis cell corpses can block the activation of CD8+T cells. In addition, ferroptosis is associated with poor prognosis of various tumors (85–87). This may pose a risk to the use of ferroptosis in cancer. On the other hand, it has been shown to be effective that building polymeric drugs were specifically ingested by cancer cells to interfere with ferroptosis and boost immunity (detailed in a later chapter). Therefore, targeting cancer cells ferroptosis should be more cautious.

## 4 CD8+ T cells are susceptible to being regulated by other immune cell ferroptosis in the TME

The TME is a dynamic system composed of cancer cells, cytokines, extracellular matrix, and immune cell subsets. The intercellular interactions affect the survival and function of cancer cells, stromal cells, T cells, and other immune cells. As the ultimate executors of tumor immunity, CD8+ T cells are also influenced by other immune cells. Therefore, other immune cell ferroptosis can alter the immune activity of CD8+ T cells in cancer (**Figure 1**; **Table 2**).

**Table 2:** Effects of other immune cells ferroptosis on CD8+ T cells.

Immune cells	Ferroptosis mechanism	effect	Refs
DCs	PPARG/PPARγ promotes RSL3-induced ferroptosis	Inhibition	(88)
TAMs	AOPCI downregulation promotes TAMs to M1 macrophages polarization via ferroptosis	Promotion	(89)
	DHA inhibits GPX4 to initiate ferroptosis and promotes TAMs to M1 macrophages polarization	Promotion	(90)
	Targeting xCT combined with PD-1 can significantly induce TAMs ferroptosis and enhance CD8+T cell activity.	Promotion	(91)
MDSCs	The polarization process of MDSCs mediated by TLR2 agonists is associated with ferroptosis	Promotion	(92)
	inhibiting ASAH2 promotes MDSCs ferroptosis	Promotion	(93)
PMN-MDSCs	Uptaking AA to induce spontaneous ferroptosis by FATP2	Inhibition	(94)
TINs	Acod1 ablation bolsters antitumor T cell immunity by inducing tins ferroptosis	Promotion	(95)
Treg	TCR/CD28 co-stimulation causes GPX4- deficient Treg expressing IL-1B and ferroptosis	Promotion	(96)

### 4.1 DCs ferroptosis

DCs are the main pathway for the activation of CD8+ T cells. DCs can recognize tumor-specific antigens to activate CD8+ T cells by TCR receptors (97). Therefore, DCs ferroptosis blocks the activation pathway of CD8+ T cells and downregulates anti-cancer immunity. Han et al. found that PPARG/PPARγ, a nuclear receptor involved in regulating lipid metabolism, promotes RSL3-induced ferroptosis of DCs. Genetic depletion of PPARG restores the maturation and function of DCs, activates cytotoxic T cells through signal transduction, and enhances CD8+ T cell-mediated anti-cancer immunity (88).

### 4.2 Macrophages ferroptosis

Tumor-associated macrophages (TAMs) play a “double-edged sword” role in the development of cancer. This may be related to TAMs subsets. Although M1-like macrophages and M2-like macrophages are TAMs subsets, M1-like macrophages showed anti-tumor immunity while M2 macrophages showed pro-tumor effect (98). Iron overload and ROS accumulation have been shown to promote the polarization of macrophages toward M1-like macrophages during ferroptosis. This may lead to partial polarization of TAMs toward M1-like macrophages and reduce the number of intra-tumoral M2-like macrophages to increase CD8+ T cell infiltration in the TME (99–103). Li et al. demonstrated that Dihydroartemisinin (DHA) increases intracellular iron levels by upregulating TFR1 and, in combination with GPX4 inhibition, initiates ferroptosis. In turn, lipid peroxidation during ferroptosis induces DNA damage response and further activates NF-kB, promoting polarization of macrophages toward the M1 phenotype (89). Similarly, Hao et al. found that inhibition of AOPCI promotes the upregulation of the multiple ferroptosis genes and enhances M2 macrophage polarization toward to M1 phenotype through the ferroptosis pathway, thereby activating CD8+ T cells and promoting anti-cancer immunity in hepatocellular carcinoma (90). Consistently, Tang et al. found that TAMs manifested xCT upregulation, ferroptosis Reduction, and M2-like polarization in hepatocellular carcinoma. Targeted xCT-mediated ferroptosis and protumoral polarization of Macrophages is effective. Inhibition or elimination of xCT combined with PD-1 can significantly reduce TAMs infiltration and M2-macrophage polarization, and enhance CD8+T cell activity (91).

### 4.3 MDSCs ferroptosis

Myeloid-derived suppressor cells (MDSCs) are immunosuppressive components of the TME. They can suppress the anti-cancer effects of CD8+ T cells. Promoting the polarization of MDSCs and reducing their infiltration can improve cancer immunotherapy (104). Li et al. found that TLR2 agonists promote the polarization of MDSCs and the production of ROS in hepatocellular carcinoma, which may be related to Runx1 in MDSCs. RNA sequencing of MDSCs after TLR2 agonist treatment revealed differential gene expression concentrating in the ferroptosis pathway, suggesting a link between MDSCs polarization and ferroptosis. Polarized MDSCs can increase CD8+ T cell activity and suppress tumor growth (92). Zhu et al. also found that ASAH2 is highly expressed in MDSCs and inhibits ferroptosis mediated by the p53 protein pathway. Therefore, inhibiting ASAH2 promotes ferroptosis in MDSCs, reducing their immunosuppressive ability and improving prognosis through CD8+ T cell infiltration and IFN-γ secretion (93). It is worth noting that the decrease in immunosuppressive cells is generally believed to alleviate the immunosuppressive effects of the TME. However, pathologically activated neutrophils, known as polymorphonuclear myeloid-derived suppressor cells (PMN-MDSCs), have more immunosuppressive effects due to spontaneous ferroptosis mediated by low oxygen and FATP2. Although ferroptosis reduces the presence of PMN-MDSCs, the release of oxygenated lipids and prostaglandin E2 (PGE2) limits the activity of CD8+ T cells in humans and mice. In immunocompetent mice, genetic or pharmacological inhibition of ferroptosis can prevent PMN-MDSCs ferroptosis and reduce their suppressive activity, slowing tumor progression. The synergistic effect with ICIs can further enhance anti-tumor immunity, opening up new perspectives on the role of ferroptosis in immunosuppressive cells (94). Previous reports verified the equivalent identity between PMN-MDSCs and tumor-infiltrating neutrophils (TINs) (105, 106). Zhao et al. identified aconitate decarboxylase 1 (Acod1) as the most upregulated metabolic enzyme in TINs. Acod1 produces itaconate through the GM-CSF-JAK/STAT5-C/EBPb pathway to defend against ferroptosis and upholds the persistence of TINs. Acod1 ablation bolsters antitumor T cell immunity and boosts the efficacy of immune checkpoint blockade by inducing TINs ferroptosis (95).

### 4.4 Treg ferroptosis

Regulatory T cell (Treg) is another important component of the immune suppression TME, which can induce CD8+ T cell exhaustion and cause the immune escape of cancer cells (107, 108). Treg evades ferroptosis by upregulating Gpx4 in the TME. TCR/CD28 co-stimulation leads to excessive accumulation of lipid peroxides and subsequent ferroptosis in Gpx4-deficient Treg, reducing the suppressive effect of Treg in the TME. In addition, IL-1B is expressed during the ferroptosis process of GPX4-deficient Treg, promoting the activation of DCs and CD8+ T cells, and inhibiting tumor growth (96).

## 5 The applications of CD8+ T cells and ferroptosis in cancer therapy

**Figure 2:**
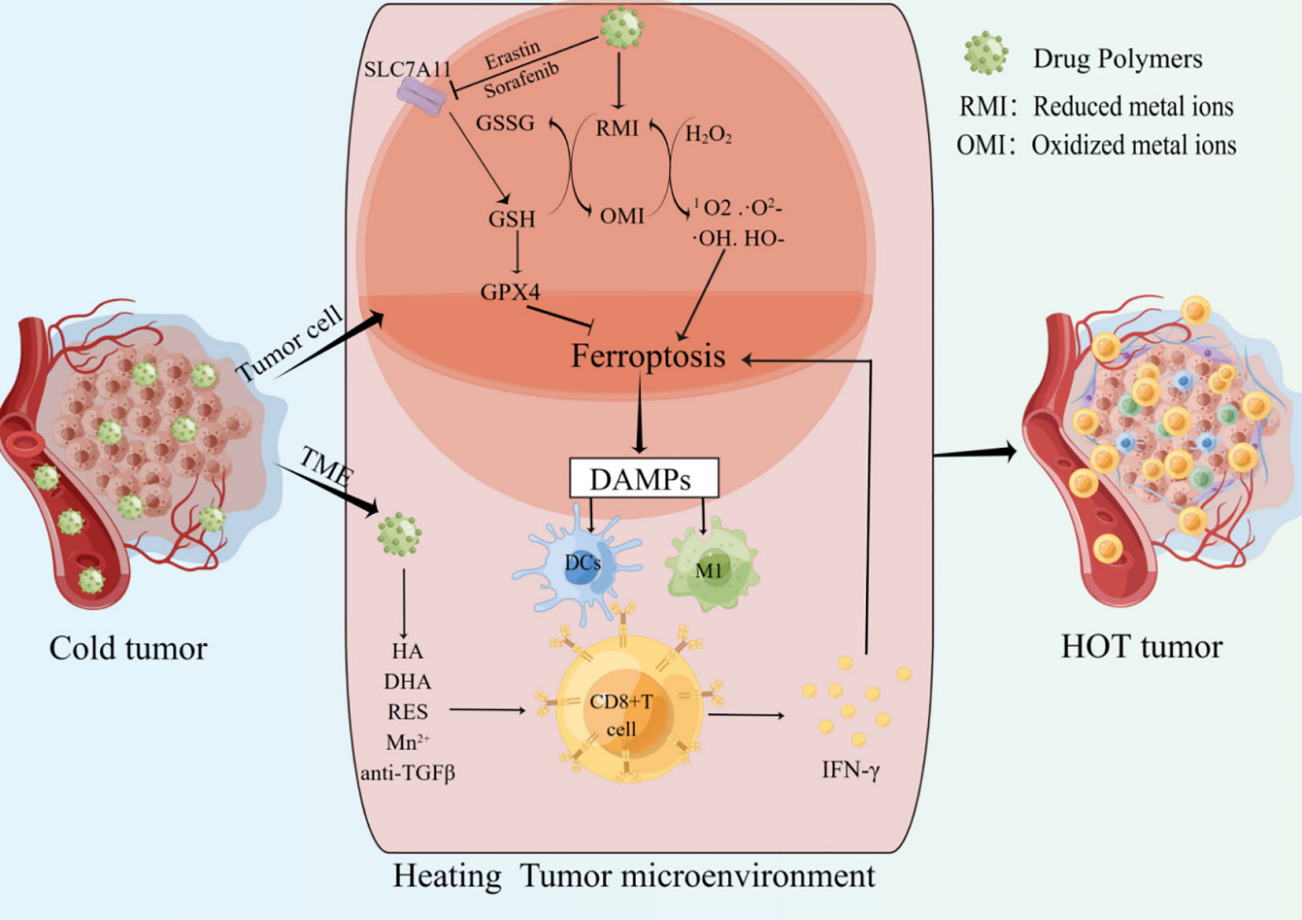
Drug polymers enter cancer cells and release metal ions or ferroptosis inducers. the shift of Metal ions between oxidized and reduced states can deplete GSH, generate highly toxic free radicals, and increase the accumulation of ROS. Ferroptosis inducers can inhibit SLC7A11 and other targets to induce tumor cells ferroptosis. The release of ferroptosis-related DAMPs promotes the infiltration and activation of CD8+ T cells. IFN-γ derived from CD8+ T cells can further promote tumor cells ferroptosis. In addition, drug polymers can also directly release drugs to promote the infiltration and activation of CD8+ T cells in the tumor microenvironment. Multiple mechanisms together heat the tumor microenvironment and improve the anti-tumor ability.

ICIs have achieved great success in the clinical application of tumor immunotherapy. However, they are ineffective in “cold tumors” lacking T cell infiltration. The strategy of converting “cold tumors” into “hot tumors” is an immunotherapy approach (**Figure 2**) (109). As mentioned above, there is a positive feedback loop promoting the relationship between CD8+ T cells and ferroptosis in cancer. Cancer cells ferroptosis can heat the TME and enhance the anti-tumor immune ability, the increased CD8+T cells can further inhibit tumor growth by promoting cancer cells ferroptosis, which may provide new hopes for cancer therapy (**Table 3**).

**Table 3:** Therapies targeting CD8+ T cells and ferroptosis.

Drug Targets	Drug-polymers	Mechanism	Refs
CD8+ T cell	GO-PEI-PEG/PD-L1siRNA	Inhibition of PD-L1-mediated CD8+ T cell depletion promotes ferroptosis in hepatocellular carcinoma cells	(110)
	SB-LC	Antigen activates CD8+ T cells via DCs to inhibit tumor growth by ferroptosis pathway	(111)
Cancer cell	IrFc1	Photoactivation causes oxidative stress and generates lipid peroxidation, leading to ferroptosis in cancer cells	(112)
	TA-Fe3+-DOX-DSPE-PEG	Increase intracellular ROS and LPO through Fenton reaction and mediate cellular ferroptosis	(113)
	PCN-Oxpt/PEG	GSH depletion, -OH production, and Fenton reaction induce ferroptosis in cancer cells	(114)
	Cu2-x Se/ZIF-8@Era-PEG-FA	Erastin and Cu2+ induce ferroptosis by inhibiting SLC7A11 and depleting GSH in cancer cells	(115)
	Ir-pbt-Bpa	Light irradiation generates singlet oxygen and superoxide anion radicals to induce ferroptosis	(116)
	CDC@SRF	Inhibition of the system XC- and direct GSH depletion significantly enhanced ferroptosis.	(117)
Combination	ZVI-NP	Triggering ferroptosis through NRF2 degradation. Improves the immune activity of CD8+ T cells.	(118)
	Fe3O4@Chl/Fe-CNPs- CPBA	Depletes GSH and promotes lipid peroxide-mediated ferroptosis. Decreases PD-L1, IDO-1, and TGF-β as well as increases CD8+ T cells.	(119)
	miR-21-3p-AuNp	Targeting TXNRD1 triggers ferroptosis and enhances the tumor-suppressive effect of ICIs.	(120, 121)
	FeCo/Fe-Co DAzyme/PL	Triggers ROS storm, GSH depletion, GPX4 inactivation, and LOX catalysis, promote ferroptosis in cancer cells and enhance IFNγ and AA action.	(122)
	Fe3+-TA@HA	Peroxidase activity and depletion of GSH induce ferroptosis and promote the recruitment of CD8+ T cells.	(123)
	RES	HMMR-SLC7A11 interaction mediates ferroptosis in cancer cells. Enhanced cytotoxic effects of CD8+ T cells co-cultured with cancer cells	(124)
	DHA	Ferroptosis was induced by the upregulation of P53 and ALOX12. CD8+ T cells were also increased in tumor tissues of DHA-treated mice	(125)
	Fe-MnO2/DHA	Promote cancer cell ferroptosis and intra-tumor CD8+ T cell immunity via DHA and Mn2+	(126)

### 5.1 Promoting CD8+ T cell-mediated cancer cells ferroptosis significantly inhibits tumor growth

Cytotoxic T-lymphocyte-associated protein 4 (CTLA-4) and programmed cell death protein 1 (PD-1) are important inhibitors of T-cell responses (127, 128). Maintaining the activation of CD8+ T cells and protecting them from exhaustion and death is an effective approach in cancer immunotherapy (129, 130). Anti-CTLA-4 antibodies can attenuate the inhibition of CD8+ T cell activity, and PD-1/PD-L1 checkpoint blockade can reduce CD8+ T cell exhaustion. Therefore, blocking CTLA-4 and PD-1/PD-L1 can inhibit CD8+ T cell exhaustion and death to promote cancer cells ferroptosis, and suppress tumor growth (131, 132).

Graphene oxide (GO) is a good drug delivery carrier, and PEI-PEG is a water-soluble polymer with high-density amine and a solubilizer that can maintain stable circulation of drug molecules in the blood (133–135). Zhao et al. developed a polymeric drug molecule (GO-PEI-PEG/PD-L1 siRNA) that carries PD-L1 siRNA and can be specifically taken up by cancer tissue. The PD-L1 siRNA is released under lysosomal action, reducing the abundance of cancer cells PD-L1 and preventing PD-L1/PD-1-mediated CD8+ T cell exhaustion. Combined therapy with sorafenib significantly improved the intra-tumoral CD8+ T cell infiltration and upregulated IFN-γ expression, promoting hepatocellular carcinoma ferroptosis (110). Besides, Huang et al. designed a porous and hollow carrier using attenuated Burkholderia pseudomallei as a vehicle, loading with tumor cells lysate and adjuvant CpG to serve as a tumor vaccine(SB-LC). Tumor-associated-antigens promoted the maturation of DCs by binding to PAR1 and MR on the surface of DCs, leading to the activation of CD8+ T cells. Enhancing the anti-cancer effect of CD8+ T cells inhibits tumor growth through the ferroptosis pathway. the positive feedback loop significantly suppressed tumor growth in various mouse tumor models (111).

### 5.2 Targeting cancer cells ferroptosis promotes the transition from “cold tumors” to “hot tumors”

High ferroptosis sensitivity of immune cells limits the non-targeted application of ferroptosis inducers in cancer (44). Therefore, it is crucial to construct targeted drugs that can be specifically taken up by cancer cells and induce ferroptosis. Multiple molecules have been shown to play important roles in the process of ferroptosis, and targeting key nodes can induce ferroptosis (136, 137). “Cold tumors” are described as lacking immunogenicity and low T cell infiltration. Cancer cell ferroptosis has been shown to promote the transition from “cold tumors” to “hot tumors” and enhance adaptive immune response (**Figure 2**) (138). One study showed that ferroptosis was a kind of ICD and promoted the maturation of BMDCs, then continuously activated the adaptive immune system and inhibited tumor growth *in vivo* (139). In addition, Incorporating ferroptosis and ultrasound-triggered sonodynamic therapy (SDT) synergistically elicited strong antitumor immunity by increasing the numbers of mature DCs and activated CD8+ cells and decreasing the number of MDSCs in the TME (140). Currently, preclinical studies about ferroptosis mainly focus on the construction of drug polymers that can be specifically taken up by cancer cells. These drug polymers release metal elements in cancer cells, and the transition between the reduced and oxidized states of these metal ions consumes GSH, including iron (Fe), manganese (Mn), copper (Cu), and iridium (Ir). Depletion of GSH increases lipid peroxidation, and this transition can also use H2O2 to generate highly toxic free radicals (Fenton reaction), such as 1O2, ·O2-, ·OH, HO-, amplify the ROS storm, promote lipid peroxidation and ferroptosis. In addition, drug polymers can also carry ferroptosis inducers, such as oxaliplatin prodrug, Erastin, and sorafenib, to promote cancer cell ferroptosis by inhibiting the intracellular system XC- and depleting GSH. Overall, regardless of how drug-polymer molecules induce cancer cell ferroptosis, the DAMPs released from cancer cell ferroptosis can activate APCs and CD8+ T cells to heat the TME and further enhance tumor suppression.

Based on the role of iron in ferroptosis, Ling et al. prepared a photosensitizer containing Fe2+ (IrFc1). IrFc1 can entered triple-negative breast cancer cells (TNBC) through transferrin, caused oxidative stress and lipid peroxidation when activated by light. This can lead to cancer cells ferroptosis and induce CD8+ T cell infiltration to enhance anti-tumor immunity (112). Jeong et al. created a polymer carrying Fe3+ (TA-Fe3- DOX-DSPE-PEG), which increased intracellular ROS and LPO through the Fenton reaction to mediate cell ferroptosis and enhance CD8+ cell-mediated anti-tumor immunity in cancer cells (113). Hu et al. prepared a polymer that not only carries Fe3+ but also adds oxaliplatin prodrug (PCN-Oxpt/PEG). The generation of oxaliplatin consumes GSH accompanied by the production of highly toxic -OH. Fe3+ further induces cancer cell ferroptosis through the Fenton reaction, and ferroptosis-related DAMPs increase CD8+ T cells derived IFN-γ to further enhance cell ferroptosis (114). Other metal ions can also promote ferroptosis in cancer cells through redox reactions. Li et al. prepared a polymer-drug molecule carrying erastin and Cu2+(Cu2-xSe/ZIF- 8@Era-PEG-FA). Erastin is released to inhibit SLC7A11/SLC3A2, and Cu2+ further enhances cancer cell ferroptosis through the mutual conversion between Cu+ and Cu2+. Reducing the miR301 in cancer cell-derived exosomes promotes the polarization of TAMs the M1 phenotype. These factors increase CD8+T cell infiltration to enhance tumor suppression (115). Ir is another metal element. Wang et al. synthesized a complex containing Ir3+ (Ir-pbt-Bpa). The intracellular Ir-pbt-Bpa can generate singlet oxygen and superoxide anion radicals under light irradiation, inducing ferroptosis and releasing DAMPs to induce CD8+ T cell immune responses. Even if only one site is irradiated, Tumors in the other site are significantly inhibited, indicating that Ir-pbt-Bpa can increase the number of effector memory T cells and achieve long-term anti-tumor immunity (116). Sorafenib is a drug approved by the FDA for the treatment of various advanced solid tumors. Zhou et al. prepared a cinnamaldehyde dimer carrying sorafenib (CDC@SRF), which rapidly ruptures in the cytoplasm after reaching the tumor, releasing drugs. SRF significantly enhances ferroptosis by inhibiting the system XC- and directly consuming GSH, and it also promotes DCs maturation and CD8+ T cell activation, triggering a strong immune response *in vivo*. After multiple doses of injection, CDC@SRF cured all mice with breast cancer (117).

### 5.3 Targeting both CD8+ T cells and cancer cell ferroptosis amplifies tumor suppression

Given the potent anti-tumor abilities of both CD8+ T cells and cancer cell ferroptosis, constructing nanopolymer drugs containing CD8+ T cell activators and ferroptosis targets can simultaneously activate CD8+ T cells and promote cancer cell ferroptosis, thereby amplifying the tumor therapeutic effects. ZVI-NP prepared by Hsieh et al. exhibits dual anti-tumor effects in cancer. The first mechanism involves the activation of the AMPK/mTOR signaling pathway, enhancingGSK3/-TrCP-dependent NRF2 degradation, thereby triggering ferroptosis in lung cancer cells. The second mechanism involves regulating TAMs to polarize toward the M1 phenotype and enhancing the immune activity of CD8+ T cells, fully exploiting the roles of ferroptosis and CD8+ T cells in cancer (118). Similarly, Chin et al. prepared CPBA-modified Fe3O4@Chl/Fe-CNPs to be ingested by targeting glycoprotein on bladder cancer cells, depleting GSH through the Fenton reaction, and promoting lipid peroxide-mediated ferroptosis with photodynamic therapy (PDT) and chemical dynamic therapy (CDT). The nanopolymer also reprograms the tumor immune microenvironment by reducing PD-L1, IDO-1, and TGF-β and increasing CD8+ T cells, M1-like macrophages, which unleashes the potential of transforming the tumor from cold tumor to hot tumor, greatly inhibiting tumor growth, and improving the survival rate of bladder cancer mice (119). Gold nanoparticles (AuNp) are excellent drug-delivery tools. Singh et al. applied AuNp loading miR-21-3p to directly target TXNRD1 in cancer cells, disrupting the redox balance and triggering ferroptosis, enhancing the sensitivity to anti-PD-1 antibodies. Therefore, the combination of miR-21-3p-AuNp and ICIs significantly enhances tumor suppression (120, 121). Liu et al. constructed a composite nano-platform that co-expresses six enzymes(FeCo/Fe-Co DAzyme/PL). The nano-platform can induce ROS storms, depleting GSH, inactivating GPX4, and catalyzing LOX to promote irreversible cancer cell immunogenic ferroptosis. IFN-γ from CD8+ T cells can interact with AA generated during the oxidative storm, further enhancing ferroptosis and overcoming current immunotherapy limitations (122). It has been demonstrated that hyaluronic acid (HA) molecules binding to cancer cells can guide lymphocytes to migrate deep into tumors to enhance the efficacy of immunotherapy (141). Therefore, Zhang et al. constructed a nanopolymer (Fe3+-TA@HA) that specifically targets CD44 overexpressed in squamous cell carcinoma (SCC), inducing cancer cell ferroptosis through peroxidase activity and GSH depletion. Fe3+-TA@HA also promotes the recruitment of CD4+ and CD8+ T cells in mouse tumors and suppresses tumor growth through cytokine secretion. ICIs further enhance the tumor suppression effect (123).

Some natural molecules have been shown to directly trigger ferroptosis and tumor immunity in cancer, and their combination with ICIs further enhances tumor suppression. Resveratrol (RES) can inhibit the interaction of HMMR and SLC7A11 to mediate cancer cell ferroptosis. RES also enhances the cytotoxicity of CD8+ T cells co-cultured with cancer cells and modulates the tumor immune microenvironment (124). Another natural molecule, Dihydroartemisinin (DHA), induces pancreatic cancer cell ferroptosis through upregulation of P53 and ALOX12-dependent mechanisms. DHA treatment also increases CD8+ T cells in mouse cancer tissues (125). Previous research has shown that local Mn2+ in the TME enhances cGAS-STING activity to promote the accumulation of CD8+ T cells and IFN-γ secretion, while Mn2+ entering cancer cells consumes GSH in the process of hydroxyl radical (·OH) generation during oxidative reactions, leading to ferroptosis (142). Therefore, Huang et al. prepared a nanopolymer Fe-MnO2/DHA loaded with Mn and DHA, which can simultaneously promote cancer cell ferroptosis and CD8+ T cell immunity in cancer, amplifying the anti-tumor effects and further inhibiting tumor progression (126).

## 6 Summary and prospect

Based on the powerful anti-cancer effects of CD8+ T cells, immune therapies represented by ICIs have greatly improved the clinical efficacy of malignant tumor treatment. However, primary or acquired resistance limits the application of these drugs in tumors (143). In recent years, we have discovered a new form of CD8+ T cell-mediated tumor growth and metastasis called ferroptosis. Although there is variability in the sensitivity of CD8+ T cells and cancer cells to ferroptosis, activating CD8+ T cells and promoting cancer cell ferroptosis synergistically amplify the tumor-suppressive effect. Furthermore, the positive feedback loop between CD8+ T cells and cancer cell ferroptosis can expand the intra-tumoral CD8+ T cells, which brings new hope for CD8+ T cells and ferroptosis in cancer immunotherapy. The intra-tumoral CD8+ T cells are also regulated by other immune cells ferroptosis, such as macrophages and Tregs, which enhance the anti-cancer effect of CD8+ T cells. In contrast, DCs’ ferroptosis inhibits the activation of CD8+ T cells. It is worth noting that the ferroptosis of MDSCs, an immune-suppressive component in the TME, does not always activate CD8+ T cells. During the ferroptosis process of PMN-MDSCs, the release of oxygen-containing lipids and PGE2 limits the activity of CD8+ T cells. New evidence also suggests that cancer cell ferroptosis is not always immunogenic cell death, which may impair the antigen presentation function of DCs and weaken the CD8+ T cell-centered anti-cancer immune response (69). Therefore, CD8+ T cells and ferroptosis form a complex network in cancer. Currently, nanotechnology is mainly used to construct polymeric molecules containing multiple drugs, which inhibit tumor growth by targeting cancer cell ferroptosis and obtaining adaptive immunity. However, the complex tumor-immunity network may bring uncertainties, requiring more comprehensive and in-depth research to clarify the communication between ferroptosis and CD8+ T cells in the TME and pave the way for clinical treatment.

## Author contributions

ZL: Writing – original draft. SZ: Writing – review & editing. KW: Writing – review & editing.

## Glossary

**Table:**

GSH	Glutathione
GPX4	Glutathione peroxidase 4
TXNRD1	Thioredoxin reductase 1
PL-OOH	Lipid peroxide
PL-OH	Lipid alcohol
TfR1	Transferrin receptor 1
IRP1	Iron regulatory protein 1
IRP2	Iron regulatory protein 2
ROS	Reactive oxygen species
ACSL4	Acyl-CoA synthetase long-chain family member 4
PUFAs	Polyunsaturated fatty acids
APCs	Antigen-presenting cells
TME	Tumor microenvironment
TNF	Tumor necrosis factor
IFNγR	IFN- γ receptor
IRF	Interferon regulatory factors
P- STATs	The phosphorylation of STATs
AA	Arachidonic acid
ICIs	Immune checkpoint inhibitors
MMP	Mitochondrial membrane potential
ICD	Immunogenic cell death
DAMPs	Damage-associated molecular patterns
HMGB1	High mobility group box 1
IREB2	Iron-responsive element-binding protein 2
BMDCs	Bone marrow-derived dendritic cells
MDSCs	Myeloid-derived suppressor cells
ATP	Adenosine triphosphate
CRT	Calreticulin
DCs	Dendritic cells
HNSCC	Head and neck squamous cell carcinoma
TAMs	Tumor-associated macrophages
DHA	Dihydroartemisinin
PMN-MDSCs	Polymorphonuclear myeloid-derived suppressor cells
PGE2	Prostaglandin E2
TINs	Tumor-infiltrating neutrophils
Acod1	Aconitate decarboxylase 1
Treg	Regulatory T cell
CTLA-4	Cytotoxic T-lymphocyte-associated protein 4
PD-1	Programmed cell death protein 1
GO	Graphene oxide
SDT	Sonodynamic therapy
SRF	Sorafenib
PDT	Photodynamic therapy
CDT	And chemical dynamic therapy
AuNp	Gold nanoparticles
SCC	Squamous cell carcinoma
